# Sample Size Estimation for Non-Inferiority Trials: Frequentist Approach versus Decision Theory Approach

**DOI:** 10.1371/journal.pone.0130531

**Published:** 2015-06-15

**Authors:** A. C. Bouman, A. J. ten Cate-Hoek, B. L. T. Ramaekers, M. A. Joore

**Affiliations:** 1 Department of Clinical Epidemiology and Medical Technology Assessment (KEMTA), Maastricht University Medical Centre, Maastricht, the Netherlands; 2 Laboratory for Thrombosis and Hemostasis, Maastricht University Medical Centre, Maastricht, the Netherlands; Yale School of Public Health, UNITED STATES

## Abstract

**Background:**

Non-inferiority trials are performed when the main therapeutic effect of the new therapy is expected to be not unacceptably worse than that of the standard therapy, and the new therapy is expected to have advantages over the standard therapy in costs or other (health) consequences. These advantages however are not included in the classic frequentist approach of sample size calculation for non-inferiority trials. In contrast, the decision theory approach of sample size calculation does include these factors. The objective of this study is to compare the conceptual and practical aspects of the frequentist approach and decision theory approach of sample size calculation for non-inferiority trials, thereby demonstrating that the decision theory approach is more appropriate for sample size calculation of non-inferiority trials.

**Methods:**

The frequentist approach and decision theory approach of sample size calculation for non-inferiority trials are compared and applied to a case of a non-inferiority trial on individually tailored duration of elastic compression stocking therapy compared to two years elastic compression stocking therapy for the prevention of post thrombotic syndrome after deep vein thrombosis.

**Results:**

The two approaches differ substantially in conceptual background, analytical approach, and input requirements. The sample size calculated according to the frequentist approach yielded 788 patients, using a power of 80% and a one-sided significance level of 5%. The decision theory approach indicated that the optimal sample size was 500 patients, with a net value of €92 million.

**Conclusions:**

This study demonstrates and explains the differences between the classic frequentist approach and the decision theory approach of sample size calculation for non-inferiority trials. We argue that the decision theory approach of sample size estimation is most suitable for sample size calculation of non-inferiority trials.

## Introduction

Non-inferiority trials that evaluate whether a new therapy is not inferior to the standard therapy, are becoming more common in the last two decades.[1,2] An important reason for conducting a non-inferiority trial is when a new therapy is expected to have advantages over the standard therapy, other than the main therapeutic effect.[3] For example, the new therapy is expected to save costs or lead to less side effects or complications. In the traditional frequentist approach of sample size calculation for non-inferiority trials, the costs and health consequences, beyond the main therapeutic effect, are not taken into consideration.[4] In this paper we argue and illustrate, using an exemplifying case study, that decision theory may provide a more comprehensive, and hence more appropriate, approach to sample size calculation for non-inferiority trials.

**Fig 1:**
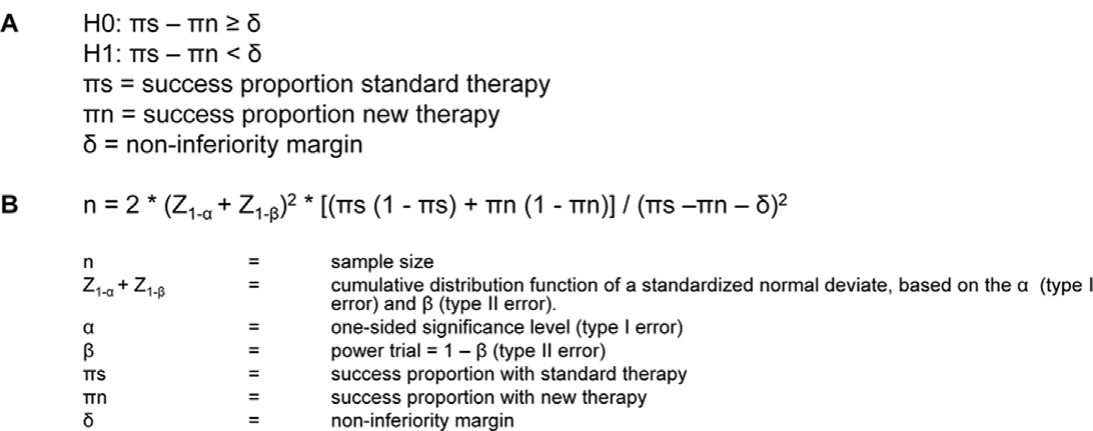
Formula Box 1.

As described in literature by Schumi et al. and Laster et al. non-inferiority trials aim to show that the new therapy is ‘not unacceptably worse’ or ‘at least as good as’ the standard therapy, with respect to the main therapeutic outcome.[3,5] It is not possible to prove equivalence of two therapies, as the null hypothesis of no difference is impossible to prove.[6] Blackwelder developed a one-sided significance test to assess whether the loss of therapeutic efficacy of the new therapy compared to the standard therapy is not more than a prespecified clinically accepted amount (Fig 1, panel A). This prespecified maximal accepted loss of therapeutic effect is called the non-inferiority margin (δ).[6] If the loss of therapeutic effect of the new therapy compared to the standard therapy does not exceed the non-inferiority margin, the new therapy is considered non-inferior to the standard therapy.[3] The sample size of a non-inferiority trial is calculated based on the non-inferiority margin, the intended power, and the significance level.[4,6] Conceptually, it is calculated what sample size is needed to prove, with statistical significance and a certain power, that the loss of therapeutic effect of the new therapy compared to the standard therapy is not larger than what is deemed maximally acceptable. An accepted difference in main therapeutic effect, the non-inferiority margin, is introduced in order to enable testing for statistical significance. Although guidelines exist on the choice of the non-inferiority margin, the choice of the margin remains arbitrary.[3,7,8]

Another approach of sample size calculation finds its origin in decision theory. In health care decision making two distinct but related decisions are made: whether or not to adopt a new therapy, and whether or not to do further research to obtain additional information.[9] In these decisions not only the main therapeutic effect, but also costs and other health consequences (such as complications, side effects and the quality of life impact associated with these health outcomes) are taken into account. The health outcomes are usually summarized in quality adjusted life years (QALYs). The expected costs and QALYs of each therapy are expressed in net monetary benefits (NMB). The therapy with the highest expected NMB should be adopted. Uncertainty surrounding the expected NMB can result in wrongful decisions and, thus, benefits forgone. Obtaining additional information can reduce uncertainty, increasing the probability of making the right decision on adoption of the new therapy.[10] The maximum value that can be ascribed to the additional information (i.e. value of information) can be compared to the costs of acquiring additional information, for example trial costs. This information can be used to estimate the optimal sample size of a trial (expected value of sampling information; EVSI).[9] The costs of acquiring additional information can be subtracted from the EVSI to obtain the value of a trial (i.e. expected net benefit of sampling; ENBS). Hence, the EVSI and ENBS analyses can be performed to determine the optimal sample size. Conceptually, it is calculated at what sample size the difference between the costs of acquiring additional information and the value of the additional information is maximum.[11–14] Several studies applied the decision theory approach to a health care decision problem.[15–23] These studies all focused on superiority trails. In our opinion, the decision theory approach of sample size estimation will be even more valuable for non-inferiority trials, because in the decision theory approach not only the main therapeutic effect, but also costs and other health consequences are taken into account. In non-inferiority trials, it often is the latter in which the new therapy is supposed to make a difference, as the main therapeutic effect is expected to be at least as good as or not unacceptably worse.

The aim of this study is to demonstrate that the decision theory approach of sample size calculation is more appropriate for non-inferiority trials than the frequentist approach of sample size calculation. To this end, the conceptual as well as the practical aspects of both approaches are compared and both approaches are applied to a case. Furthermore, we aim to initiate discussion on this topic of sample size calculation for clinical trials, because there is need for innovation.

## Materials and Methods

### Frequentist approach

The sample size needed to prove non-inferiority of a new therapy was calculated using the formula shown in Fig 1, panel B. The non-inferiority margin, success proportion of the standard therapy and the new therapy, and the intended significance level (α) and power (1 - β) of the trial were specified, subsequently the sample size was calculated.[4,6]

### Decision theory approach

**Fig 2:**
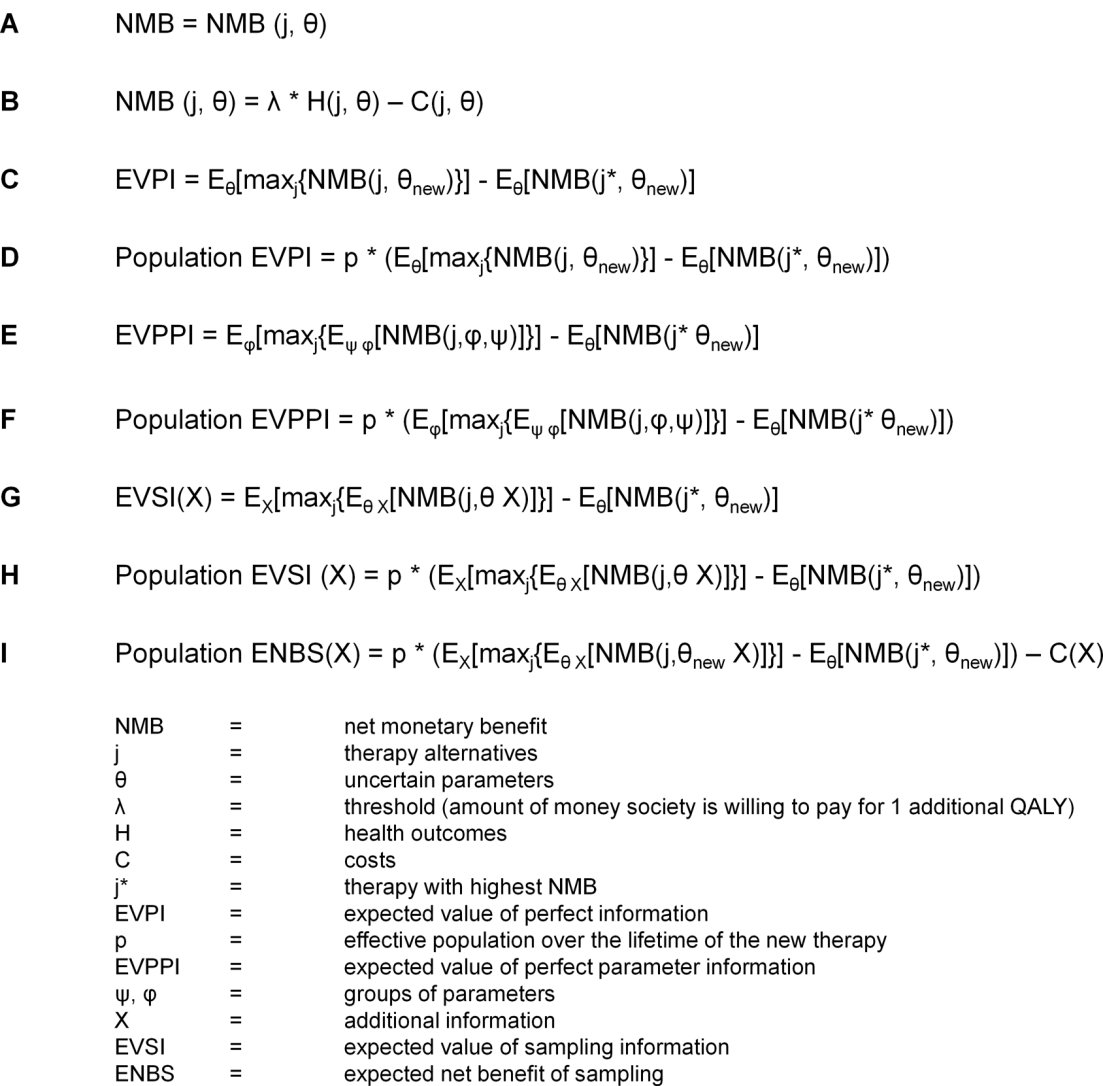
Formula Box 2.

Health economic decision modeling can be used to assess the relevant effects and costs of a new therapy against its comparator over time. Whether or not to adopt a new therapy depends on the health effects and the costs of the new therapy. A health care decision model can be used to estimate the costs and health effects of the therapy alternatives **j**, given uncertain parameters represented as vector **θ** (Fig 2, panel A).[24] Every value of the uncertain parameters **θ**, gives different costs (C(j, θ)) and health outcomes (H(j, θ)). The expected NMB is the difference in health outcomes of the different therapy alternatives times the threshold (amount of money society is willing to pay for additional health outcomes **λ**), minus the difference in costs (Fig 2, panel B). The therapy with the highest NMB, is the therapy of choice (j*). Obtaining new information can reduce the uncertainty on some or all uncertain parameters **θ**_**new**_. When perfect information would be available (no uncertainty) on all parameters, the right decision would always be made and the therapy with the highest NMB would always be chosen. Thus, the value of having perfect information on **θ**_**new**_, is the amount of money that could be saved if the therapy with the highest NMB is always chosen. This is called the expected value of perfect information per patient (EVPI) (Fig 2, panel C).[10] The number of patients that is affected by the decision to adopt the new therapy over the lifetime of the new therapy is called the effective population, represented as **p**. The effective population is used to calculate the EVPI for the whole population involved (Fig 2, panel D). Further research usually cannot address all uncertainty as studies obtain information on only one or a few uncertain parameters. Therefore, it is useful to calculate the expected value of perfect information on a certain parameter or a certain group of parameters (θ_new_ = (φ,ψ)), the expected value of perfect parameter information (EVPPI) (Fig 2, panel E and F). Studies will have to be performed to obtain additional information, represented as **X**, on a parameter or a group of parameters. After obtaining additional information **X** on parameters **θ**, the posterior probability distribution of **θ** will have smaller confidence intervals. The more the reduction in uncertainty is, the higher the value of the additional information. It is possible to calculate the expected value of information for different samples sizes of a study. This is called the expected value of sampling information (EVSI) (Fig 2, panel G and H).[10] Performing studies costs money: C(X). Therefore, the net gain of obtaining additional information is the expected value of sampling information minus the costs made. This is called the expected net benefit of sampling (ENBS) (Fig 2, panel I).[12]

To estimate the optimal sample size according to the decision theory approach, a health economic decision model was build and EVSI analyses were performed. For different sample sizes, possible trial results were simulated multiple times, using Monte Carlo simulation. According to Bayesian methods, model parameters were updated with the simulated trial results, and an updated EVPI per patient was calculated. To calculate the updated population EVPI, the sample size of the trial was subtracted from the effective population, because the patients participating in the trial do not benefit from the additional information obtained in the trial. The EVSI is the prior population EVPI (before update with simulated trial results) minus the updated population EVPI. The EVSI was calculated for different sample sizes.[9] Costs of the trial were defined and subsequently the ENBS was calculated to assess for which sample size the net gain was optimal. Sensitivity analyses were performed to explore the effect of changes in the size of the effective population on the EVSI and ENBS results.

In Table 1 the most essential differences between the frequentist approach and the decision theory approach of sample size calculation for non-inferiority trials are described.

**Table 1:** Conceptual and practical aspects of the frequentist and the decision theory approach of sample size estimation for non-inferiority trials.

*Characteristics*	*Frequentist approach*			*Decision theory approach*		
Aim	Determine what the sample size of a future trial should be to prove, with statistical significance and a certain power, that the loss of therapeutic effect of the new therapy compared to the standard therapy is less than the non-inferiority margin.			Determine at what sample size of a future trial the difference between the costs of acquiring additional information and the societal value of the additional information is maximum.		
Analytical approach	Formula			EVSI and ENBS analyses using a probabilistic health economic decision model		
*Parameters*	*In?*	*Parameter estimate*	*REF*	*In?*	*Parameter estimate*	*REF*
**Main therapeutic outcome**						
Success proportion standard therapy	Yes	23.3%*	[26]	Yes	21.1% (SE 0.0429), 22.2% (SE 0.0431), 24.5% (SE 0.0454)^#^	[26]
Success proportion new therapy	Yes	Equal to success proportion standard therapy	[33]	Yes	Success proportion standard therapy x Relative risk parameter	[33]
One-sided significance level	Yes	5%		No		
Power	Yes	80%		No		
**Difference main therapeutic effect**						
Non-inferiority margin	Yes	7.5%	[26,33] e.o.	No		
Relative risk parameter	No			Yes	1.000 (95% CI 1.000–3.316)	[26,33] e.o.
**Costs and other health consequences**						
Resource use associated with treatment and consequences	No			Yes	Estimates of resource use (stockings, homecare for application of stockings, treatment of PTS)^&^	[30,46–48]
Unit prices	No			Yes	Unit prices of resource use ^&^	[49]
Quality of life consequences	No			Yes	Post DVT no PTS: age dependent norm utility, disutility mild to moderate PTS: 0.117 (SE 0.050), disutility severe PTS: 0.218 (SE 0.040)^&^	[50,51]
Time horizon	Yes	2 years	[26]	Yes	Lifetime	[52]
Discount rate	No			Yes	Costs: 0.04, effects: 0.015	[38]
Threshold for a unit of effect	No			Yes	€ 20,000 per QALY	[39]
**Value for society**						
Costs of research	No			Yes	€ 10,000 fixed, € 5000 per included patient	e.o.
Lifetime new therapy	No			Yes	10 years	e.o.
Annual incidence	No			Yes	25,000 patients	[29]
Sample size	788			500		

*ARR PTS after 2 years,

^#^Cumulative incidence PTS after 6, 12, and 24 months respectively,

^&^For details see appendix.

ARR, absolute risk reduction; ENBS, expected net benefit of sampling; e.o., expert opinion; EVSI, expected value of sampling information; PTS, post thrombotic syndrome; QALY, quality adjusted life year.

### Case description

Twenty to fifty percent of the patients who suffer from a DVT of the leg, develop PTS.[25,26] Patients with PTS have complaints of the leg that was affected by the DVT; for example pain, heaviness, cramps, tingling, itching, and in severe cases ulceration of the leg.[25,26] Patients with mild to moderate PTS report a quality of life that is lower than contemporaries with arthritis, chronic lung disease, hearing impairment, or diabetes. Patients with severe PTS report a quality of life that is comparable to patients with angina, cancer, or congestive heart failure.[27] The healthcare costs of PTS were found to be $7000 per patient per year, in a retrospective study in the USA on administrative claim data of patients after DVT.[28] There is no effective treatment for PTS, and prevention remains the cornerstone for disease management. Two large randomized controlled trials (RCT) showed a relative risk reduction in PTS of approximately 50% when patients wore elastic compression stockings (ECS) for a period of two years after DVT.[25,26] ECS are not very costly, roughly 100 euro per patient per year. However, the incidence of DVT is substantial. Every year, 25.000 patients develop PTS in the Netherlands [29], and around 7.5% [30] of the patients need home care to apply and take off the stockings (€ 10.000 euro per patient per year [31]). As a result, in the Netherlands the total annual impact on the health care budget amounts to approximately 23.5 million euro. Besides the cost issue, compliance is a major problem of ECS therapy. Patients do not wear the stocking because it is warm, itching, and pinching. Elderly patients are restricted in their freedom of movement because they have to wait at home until the nurse arrives to apply or take off their stocking. In the end, more than 50% of patients do not develop PTS.[32] A new strategy of ECS therapy is to tailor the duration of therapy on the signs and symptoms of PTS of the individual patient, after the first six months of therapy. In a management study including 125 patients, this approach was safe and effective, as the incidence of PTS was comparable to the PTS incidence in the active arm of the two previous RCTs.[33] In addition, approximately 50% of patients could safely take off the stocking after 6 months.[33] This strategy appears to be safe and potentially cost saving. However, to confirm the results of the management study, a randomized non-inferiority trial should be performed to assess whether individually tailored ECS therapy is not inferior to two years ECS therapy, in preventing PTS.[34]

## Results

### Frequentist approach

**Fig 3:**
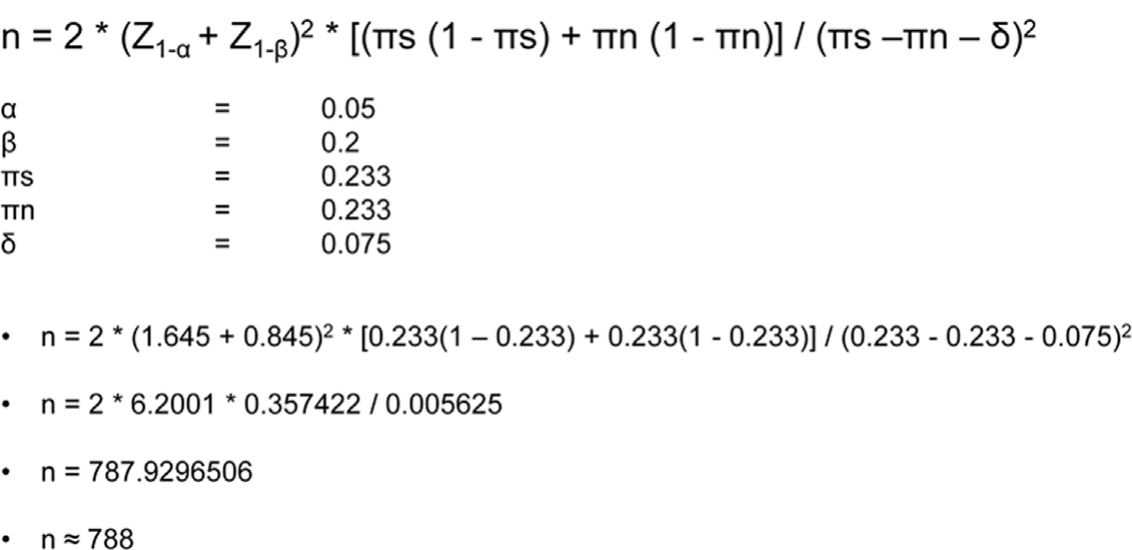
Calculations Box 1.

The non-inferiority margin was based on the proportion of therapeutic effect of the active control that should be retained.[8] In this case the active control was two years ECS therapy. The therapeutic effect of the active control was derived from an RCT comparing two years ECS therapy versus no ECS therapy, for the prevention of PTS, performed by Prandoni et al. This study found an absolute risk reduction of 23.3% of PTS at two years after DVT.[26] The non-inferiority margin was set at 7,5% in order for individually tailored ECS therapy (the new therapy) to preserve approximately 70% of the therapeutic effect of the active control. It is customarily accepted in non-inferiority trials to preserve at least 50–70% of the therapeutic effect of the active control.[35–37] Based on the management study, it was hypothesized that individually tailored ECS therapy would have an equal success proportion as two years ECS therapy.[33] At a one sided significance level of 5% and a power of 80%, a sample size of 788 needed to test the hypothesis was calculated. (Fig 3, Table 1).

### Decision theory approach

A probabilistic state transition health economic decision model was developed to assess the lifetime costs and health consequences (the main therapeutic effect, complications, side effects, and the quality of life impact associated with these health outcomes) of individually tailored ECS therapy versus two years ECS therapy in patients after DVT. The decision model consisted of four health states: No PTS, Mild to moderate PTS, Severe PTS, and Death. The difference between the two therapies was modeled by taking into account the differential impact on the probability of developing PTS, the costs of ECS therapy (for stockings and home care for stocking application), and the disutility of ECS therapy. Cycle length was 6 months for the first two cycles, and 1 year for the cycles thereafter. Cohort simulation was used to evaluate the decision model. Future costs and effects were discounted using a discount rate of 4% for costs and 1.5% for (quality adjusted) life years, in concordance with Dutch guidelines.[38]

In the decision model, PTS incidence was hypothesized to be equal for both therapies. This assumption was based on the management study.[33] The transition probabilities for developing PTS in the two years after DVT were derived from the RCT by Prandoni et al. for both therapies.[26]

A relative risk (RR) parameter was incorporated in the decision model to represent the uncertainty of the development of PTS with individually tailored ECS therapy. A log normal distribution, with a mean of 1.000 and 95% confidence intervals (CI) ranging between 1.000 and 3.316 (the standard error of the LN(RR) is 0.612) is used. The RR parameter is multiplied with the probabilities of developing PTS after six months with individually tailored ECS therapy. The upper limit of the 95% CI corresponds with a 7.5% increase in the two year PTS incidence, representing the expected maximal increase in PTS incidence with individually tailored ECS therapy. Values of the parameter below 1 are rounded up to 1, because we assume that individually tailored ECS therapy does not lead to a decrease in PTS incidence. The RR parameter is based on the same evidence as the non-inferiority margin of the frequentist approach. However, it represents the uncertainty of developing PTS with individually tailored ECS therapy, which is conceptually different from the non-inferiority margin. The decision model was built in Microsoft Excel 2010. (For more details on the decision model construction, see the Appendix.)

EVPI and EVPPI were calculated for an effective population of 25,000 patients, the yearly incidence of DVT in the Netherlands [29], and a lifetime of individually tailored ECS therapy of 10 years. The threshold was set at € 20,000 per QALY.[39] The EVSI of a non-inferiority trial comparing individually tailored ECS therapy with two years ECS therapy for the prevention of PTS, was calculated for different sample sizes (n = 25, n = 100, n = 400, n = 500, n = 700, n = 1000, n = 1500, n = 5000). The model parameters concerning the development of PTS with both individually tailored ECS therapy and two years ECS therapy, and the relative risk parameter were updated with the simulated trial results. Two hundred possible trial results were simulated using Monte Carlo simulation with 1000 iterations. For the trial we assumed a fixed cost of € 10.000,- and a variable cost of € 5000,- per included patient.

**Fig 4:**
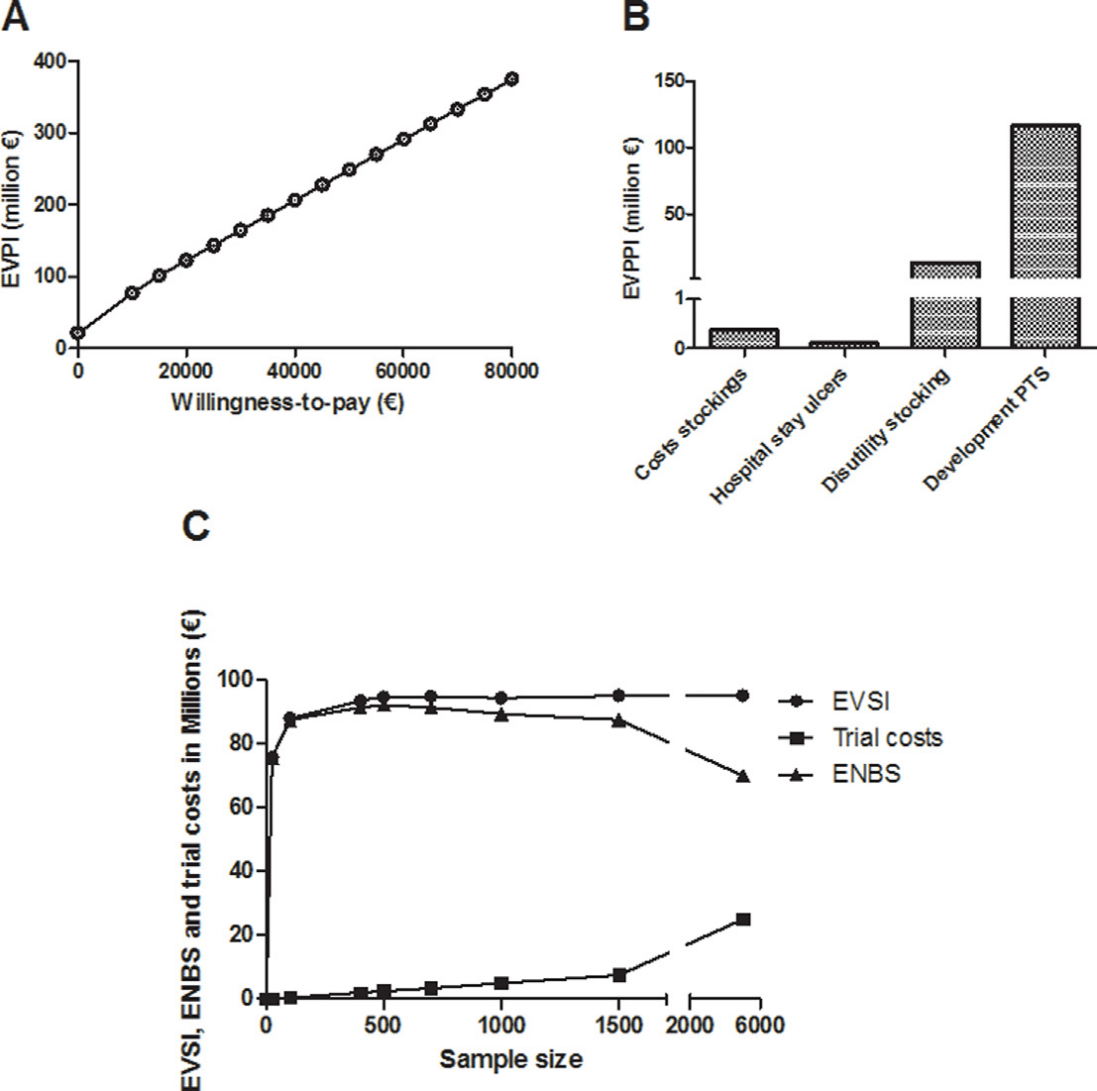
Results Value of information analyses. A)EVPI, B)EVPPI, C)EVSI and ENBS.

Based on current evidence the total expected lifetime costs were € 14,400 (95%CI 5,700–28,100) for two years ECS therapy and € 14,300 (95%CI 5,500–28,100) for individually tailored ECS therapy. The lifetime health outcomes yielded 12.50 (95%CI 11.70–13.29) QALY and 12.49 (95%CI 11.63–13.30) QALY, respectively. The differences in health outcomes and costs are small according to these analyses. Since there is a large amount of uncertainty it is worthwhile to perform a trial. The uncertainty surrounding the cost-effectiveness of individually tailored ECS therapy versus two years ECS therapy resulted in an EVPI of € 600 per patient, and the population EVPI was found to be € 122 million (Fig 4). The partial EVPI was highest for the uncertainty of the parameters concerning the incidence of PTS when applying individually tailored ECS therapy, EVPPI of € 117 million (Fig 4). The EVSI increased with an increasing sample size. However, from a sample size of 500 patients the EVSI was found to remain stable around € 94.5 million (Fig 4). The optimal sample size was found to be 500 patients (Table 1), as the ENBS reached its maximum (€ 92 million) for 500 patients (Fig 4). The sensitivity analysis revealed that the optimal sample size remained to be 500 patients for effective populations of 10,000 and 40,000, with ENBS of € 35,5 million and € 150,- million, respectively.

## Discussion

The aim of this study was to compare the conceptual and practical aspects of the frequentist approach and decision theory approach of sample size calculation for non-inferiority trials, thereby demonstrating that the decision theory approach is more appropriate for sample size calculation of non-inferiority trials. The approaches differ with respect to conceptual background, analytical approach, and input requirements. The application to the case revealed that the sample size of a non-inferiority trial comparing individually tailored ECS therapy with two years ECS therapy for the prevention of PTS after DVT was 788 patients according to the frequentist approach and 500 patients according to the decision theory approach.

With regard to the conceptual background, the two approaches take a distinctly different point of view. While according to the frequentist approach the sample size that is necessary to prove non-inferiority with statistical significance is calculated, in the decision theory approach it is calculated for what sample size value of information is optimal. A number of differences in analytical approach and input requirements result from this difference in conceptual background.

There are several differences in the analytical approach. Firstly, there is a difference in the type of statistics employed to analyse the trial data. As the sample size calculation according to the frequentist approach is based on frequentist statistics; after completion of the trial the data will be analysed using frequentist statistics. In the decision theory approach a probabilistic health economic decision model is used for the sample size calculation, and consequently after completion of the trial the data will need to be analysed with a probabilistic health economic decision model. Although outcomes in health economic decision modelling are mainly composite outcomes, it is also possible to derive singular outcomes, such as the main therapeutic effect, from the analyses. Furthermore, it is also possible to exclude costs from the analyses of composite outcomes.[40] However, analyses of composite outcomes including all relevant parameters are usually more informative. Secondly, the two approaches include the (difference in) main therapeutic effect between the two therapies in a different manner. In the frequentist approach, a pre-specified maximal accepted therapeutic effect loss is introduced to enable statistical testing. In the decision theory approach, a parameter indicating the uncertainty on the difference in main therapeutic outcome between the two therapies is applied. In our opinion, the method used in the decision theory approach is the better way of taking into account the expected difference in main therapeutic effect. Rather than a fixed difference, an estimate with assigned distribution is used, which yields a more accurate reflection of the uncertainty associated with the difference in main therapeutic outcome between the two therapies. Thirdly, the approaches deal differently with the uncertainty present. The frequentist approach includes the point estimate of the main therapeutic outcome, and uncertainty is reflected by the one-sided significance level and power. In order to reflect uncertainty in the decision theory approach, distributions are assigned to all estimates of the parameter to model the main therapeutic outcome. We feel estimates with accompanying distributions give a more genuine representation of the uncertainty, as they describe the boundaries within which the true value lies based on all current evidence.

Input requirements differ distinctively between the two approaches. Firstly, costs and health consequences, other than the main therapeutic effect, are typically not included in the frequentist approach of sample size calculation. Consequently, the frequentist sample size of non-inferiority trials can be underpowered to show a difference in the costs and other health consequences. In the decision theory approach of sample size calculation these costs and other health consequences are taken into account. Secondly, aspects of patient preferences are included in the decision theory approach of sample size calculation by inclusion of quality of life consequences associated with health states and therapies.[41] It is increasingly advocated to incorporate patient preferences in clinical guidelines and healthcare policy decisions.[41,42] In the frequentist approach of sample size estimation, aspects of patient preferences can only be included to the extent that this is reflected in the main therapeutic outcome. Therefore, only if a patient reported outcome (for example generic quality of life) is the main therapeutic outcome measure, patient preferences are involved. However, most clinical trials have clinical effect estimates (survival, recurrence) as the main therapeutic outcome.

Thirdly, the value of research for society is incorporated in the decision theory approach and not in the frequentist approach. Performing a clinical trial is a large investment of, usually, limited public financial resources. Researchers are obliged to use these financial resources sensibly, by prioritizing research activities and designing clinical trials conscientiously. The decision theory approach of sample size calculation is based on the economic principles of resource allocation, and therefore forms a framework of decision making based on the costs and consequences of all factors and consequences involved. Moreover, it appears unethical to expose patients to randomisation in a clinical trial that has not clearly assessed its future value for society a priori.

The decision theory approach of sample size calculation is far from implemented regularly in clinical research. The frequentist approach remains the most used and therefore most accepted method of sample size calculation for non-inferiority trials. A true paradigm shift is needed to allow for the universal implementation of the decision theory approach. The reality is that scientists today are condemned to participate in competitive grant writing rounds and are judged by citation rates and impact factors, and are thereby chained to adhere to what is most accepted, in order to get their work funded or published. This hampers innovation and progress.

Besides a change in mind-set, some more practical issues demand attention before universal implementation of the decision theory approach can occur. The presumed lack of acceptability of the decision theory approach may result from a number of factors. Firstly, a probabilistic health economic decision model needs to be developed and comprehensive analyses need to be performed for the sample size calculation according to the decision theory approach. This is far more time-consuming than the frequentist sample size calculation. However, the time used to consult the existing literature to estimate all model parameters (i.e. treatment effects, costs of treatment, health consequences, trial costs) and their distributions seems an appropriate investment in the planning of, often very costly, RCTs. Moreover, the decision model built can be used for (cost-effectiveness) analyses at a later stage. Secondly, the technical quality of the model, as well as the appropriateness of the model structure, data sources, assumptions, and uncertainty incorporated in the model, influence the validity of the model, and hence the sample size calculation.[43] It is crucial that the decision model is transparent and thoroughly validated. For a thorough validation of the model, several forms of validation should be addressed. 1) Face validity: clinical experts in the field should judge whether the model structure, data sources, problem formulation, and results fit in with the current science and evidence. 2) Internal validity: the mathematical calculations and consistency of the model should be checked and assessed, by i.e. elaborate verifications and sensitivity analyses. 3) Cross-validation: whenever possible the model should be compared to other models which are designed to answer the same question. 4&5) External validation and predictive validity: if feasible the results of the model should be compared to real-world data, for example the results of a clinical trial.[43] The field made efforts to harmonize the methods used in cost-effectiveness modelling by making guidelines for validation.[43,44] Of course, assumptions regarding for instance the non-inferiority margin, also influence the sample size estimation in the frequentist approach. However, as the decision theory approach uses much more inputs, the validity may be more easily violated, if only in perception. If so-called reference models for distinct disease areas would become freely available to researchers, this could eliminate these drawbacks. Finally, a disadvantage of the decision theory approach is that it is necessary to define a threshold: the maximum amount society is willing to pay for additional health outcomes, usually expressed in QALYs. Such a threshold is much-debated, and defining this threshold is not straightforward.[45] It does however assist transparent health care policy decision making.

Despite these practical drawbacks, we argue that for non-inferiority trials the decision theory approach of sample size estimation is more appropriate than the classic frequentist approach, and we hope to initiate discussion on this important topic.

## Supporting Information

S1 Appendix Detailed description of health economic decision model and analyses. (DOCX)

## References

[1] Julious SA, Owen RJ (2011) A comparison of methods for sample size estimation for non-inferiority studies with binary outcomes. Stat Methods Med Res 20: 595–612. 10.1177/0962280210378945 20889572

[2] Soonawala D, Middelburg RA, Egger M, Vandenbroucke JP, Dekkers OM (2010) Efficacy of experimental treatments compared with standard treatments in non-inferiority trials: a meta-analysis of randomized controlled trials. Int J Epidemiol 39: 1567–1581. 10.1093/ije/dyq136 20837637

[3] Schumi J, Wittes JT (2011) Through the looking glass: understanding non-inferiority. Trials 12: 106 10.1186/1745-6215-12-106 21539749 PMC3113981

[4] Laster LL, Johnson MF, Kotler ML (2006) Non-inferiority trials: the 'at least as good as' criterion with dichotomous data. Stat Med 25: 1115–1130. 16381070 10.1002/sim.2476

[5] Laster LL, Johnson MF (2003) Non-inferiority trials: the 'at least as good as' criterion. Stat Med 22: 187–200. 12520556 10.1002/sim.1137

[6] Blackwelder WC (1982) "Proving the null hypothesis" in clinical trials. Control Clin Trials 3: 345–353. 7160191 10.1016/0197-2456(82)90024-1

[7] (2006) Committee for Medicinal Products for Human Use (CHMP) guideline on the choice of the non-inferiority margin. Stat Med 25: 1628–1638. 16639773 10.1002/sim.2584

[8] Rothmann MD, Tsou HH (2003) On non-inferiority analysis based on delta-method confidence intervals. J Biopharm Stat 13: 565–583. 12921402 10.1081/BIP-120022775

[9] Eckermann S, Willan AR (2007) Expected value of information and decision making in HTA. Health Econ 16: 195–209. 16981193 10.1002/hec.1161

[10] Briggs A, Claxton K, Sculpher M (2006) Efficient research design In: Press OU, editor. Decision Modelling for Health Economic Evaluation. Oxford.

[11] Ades AE, Lu G, Claxton K (2004) Expected value of sample information calculations in medical decision modeling. Med Decis Making 24: 207–227. 15090106 10.1177/0272989X04263162

[12] Claxton K, Posnett J (1996) An economic approach to clinical trial design and research priority-setting. Health Econ 5: 513–524. 9003938 10.1002/(SICI)1099-1050(199611)5:6<513::AID-HEC237>3.0.CO;2-9

[13] Detsky AS (1985) Using economic analysis to determine the resource consequences of choices made in planning clinical trials. J Chronic Dis 38: 753–765. 3928673 10.1016/0021-9681(85)90118-3

[14] Lindley DV (1997) The choice of sample size. Statistician: 129–138.

[15] Groot Koerkamp B, Nikken JJ, Oei EH, Stijnen T, Ginai AZ, Hunink MG (2008) Value of information analysis used to determine the necessity of additional research: MR imaging in acute knee trauma as an example. Radiology 246: 420–425. 10.1148/radiol.2462070093 18227539

[16] Grutters JP, Abrams KR, de Ruysscher D, Pijls-Johannesma M, Peters HJ, Beutner E, et al (2011) When to wait for more evidence? Real options analysis in proton therapy. Oncologist 16: 1752–1761. 10.1634/theoncologist.2011-0029 22147003 PMC3248774

[17] Kent S, Briggs A, Eckermann S, Berry C (2013) Are value of information methods ready for prime time? An application to alternative treatment strategies for NSTEMI patients. Int J Technol Assess Health Care 29: 435–442. 10.1017/S0266462313000433 24290337 PMC3846382

[18] Koerkamp BG, Spronk S, Stijnen T, Hunink MG (2010) Value of information analyses of economic randomized controlled trials: the treatment of intermittent claudication. Value Health 13: 242–250. 10.1111/j.1524-4733.2009.00656.x 19818058

[19] McKenna C, Claxton K (2011) Addressing adoption and research design decisions simultaneously: the role of value of sample information analysis. Med Decis Making 31: 853–865. 10.1177/0272989X11399921 21393558

[20] Retel VP, Grutters JP, van Harten WH, Joore MA (2013) Value of research and value of development in early assessments of new medical technologies. Value Health 16: 720–728. 10.1016/j.jval.2013.04.013 23947964

[21] Thariani R, Henry NL, Ramsey SD, Blough DK, Barlow B, Gralow JR, et al (2013) Is a comparative clinical trial for breast cancer tumor markers to monitor disease recurrence warranted? A value of information analysis. J Comp Eff Res 2: 325–334. 10.2217/cer.13.15 24236631 PMC4018420

[22] Welton NJ, Madan J, Ades AE (2011) Are head-to-head trials of biologics needed? The role of value of information methods in arthritis research. Rheumatology (Oxford) 50 Suppl 4: iv19–25. 21859701 10.1093/rheumatology/ker242

[23] Willan AR (2007) Clinical decision making and the expected value of information. Clin Trials 4: 279–285. 17715257 10.1177/1740774507079237

[24] Buxton MJ (2006) Economic evaluation and decision making in the UK. Pharmacoeconomics 24: 1133–1142. 17067197 10.2165/00019053-200624110-00009

[25] Brandjes DP, Buller HR, Heijboer H, Huisman MV, de Rijk M, Jagt H, et al (1997) Randomised trial of effect of compression stockings in patients with symptomatic proximal-vein thrombosis. Lancet 349: 759–762. 9074574 10.1016/S0140-6736(96)12215-7

[26] Prandoni P, Lensing AW, Prins MH, Frulla M, Marchiori A, Bernardi E, et al (2004) Below-knee elastic compression stockings to prevent the post-thrombotic syndrome: a randomized, controlled trial. Ann Intern Med 141: 249–256. 15313740 10.7326/0003-4819-141-4-200408170-00004

[27] Kahn SR, Shbaklo H, Lamping DL, Holcroft CA, Shrier I, Miron MJ, et al (2008) Determinants of health-related quality of life during the 2 years following deep vein thrombosis. J Thromb Haemost 6: 1105–1112. 10.1111/j.1538-7836.2008.03002.x 18466316

[28] MacDougall DA, Feliu AL, Boccuzzi SJ, Lin J (2006) Economic burden of deep-vein thrombosis, pulmonary embolism, and post-thrombotic syndrome. Am J Health Syst Pharm 63: S5–15. 17032933 10.2146/ajhp060388

[29] Naess IA, Christiansen SC, Romundstad P, Cannegieter SC, Rosendaal FR, Hammerstrom J (2007) Incidence and mortality of venous thrombosis: a population-based study. J Thromb Haemost 5: 692–699. 17367492 10.1111/j.1538-7836.2007.02450.x

[30] Gelderblom GJ, Hagedoorn-Meuwissen EAV (2005) Kousen uittrekhulpmiddel Easy-Lever. Een onderzoek naar bruikbaarheid, effecten en belemmeringen, in opdracht van ZonMw.

[31] Blattler W (1999) Aspects of cost effectiveness in therapy of acute leg/pelvic vein thrombosis. Wien Med Wochenschr 149: 61–65. 10454937

[32] Kahn SR, Shrier I, Julian JA, Ducruet T, Arsenault L, Miron MJ, et al (2008) Determinants and time course of the postthrombotic syndrome after acute deep venous thrombosis. Ann Intern Med 149: 698–707. 19017588 10.7326/0003-4819-149-10-200811180-00004

[33] Ten Cate-Hoek AJ, Ten Cate H, Tordoir J, Hamulyak K, Prins MH (2010) Individually tailored duration of elastic compression therapy in relation to incidence of the postthrombotic syndrome. J Vasc Surg 52: 132–138. 10.1016/j.jvs.2010.01.089 20385462

[34] Ten Cate-Hoek AJ, Bouman AC, Joore MA, Prins M, Ten Cate H (2014) The IDEAL DVT study, individualised duration elastic compression therapy against long-term duration of therapy for the prevention of post-thrombotic syndrome: protocol of a randomised controlled trial. BMJ Open. 10.1136/bmjopen-2014-005265 PMC4158195 25190617

[35] Buller HR, Cohen AT, Davidson B, Decousus H, Gallus AS, Gent M, et al (2007) Idraparinux versus standard therapy for venous thromboembolic disease. N Engl J Med 357: 1094–1104. 17855670 10.1056/NEJMoa064247

[36] Buller HR, Gallus AS, Pillion G, Prins MH, Raskob GE (2012) Enoxaparin followed by once-weekly idrabiotaparinux versus enoxaparin plus warfarin for patients with acute symptomatic pulmonary embolism: a randomised, double-blind, double-dummy, non-inferiority trial. Lancet 379: 123–129. 10.1016/S0140-6736(11)61505-5 22130488

[37] Schulman S, Kearon C, Kakkar AK, Schellong S, Eriksson H, Baanstra D, et al (2013) Extended use of dabigatran, warfarin, or placebo in venous thromboembolism. N Engl J Med 368: 709–718. 10.1056/NEJMoa1113697 23425163

[38] Oostenbrink JB, Koopmanschap MA, Rutten FF (2002) Standardisation of costs: the Dutch Manual for Costing in economic evaluations. Pharmacoeconomics 20: 443–454. 12093300 10.2165/00019053-200220070-00002

[39] Casparie AF, van Hout BA, Simoons ML (1998) [Guidelines and costs]. Ned Tijdschr Geneeskd 142: 2075–2077. 9856218

[40] Claxton K, Griffin S, Koffijberg H, McKenna C (2013) Expected health benefits of additional evidence: Principles, methods and applications. CHE Research Paper 83. Centre for Health Economics.

[41] Krahn M, Naglie G (2008) The next step in guideline development: incorporating patient preferences. JAMA 300: 436–438. 10.1001/jama.300.4.436 18647988

[42] Boivin A, Currie K, Fervers B, Gracia J, James M, Marshall C, et al (2010) Patient and public involvement in clinical guidelines: international experiences and future perspectives. Qual Saf Health Care 19: e22 10.1136/qshc.2008.031823 20427302

[43] Eddy DM, Hollingworth W, Caro JJ, Tsevat J, McDonald KM, Wong JB (2012) Model transparency and validation: a report of the ISPOR-SMDM Modeling Good Research Practices Task Force—7. Value Health 15: 843–850. 10.1016/j.jval.2012.04.012 22999134

[44] Briggs AH, Weinstein MC, Fenwick EA, Karnon J, Sculpher MJ, Paltiel AD (2012) Model parameter estimation and uncertainty: a report of the ISPOR-SMDM Modeling Good Research Practices Task Force—6. Value Health 15: 835–842. 10.1016/j.jval.2012.04.014 22999133

[45] Smith RD, Richardson J (2005) Can we estimate the 'social' value of a QALY? Four core issues to resolve. Health Policy 74: 77–84. 16098414 10.1016/j.healthpol.2004.12.009

[46] Caprini JA, Botteman MF, Stephens JM, Nadipelli V, Ewing MM, Brandt S, et al (2003) Economic burden of long-term complications of deep vein thrombosis after total hip replacement surgery in the United States. Value Health 6: 59–74. 12535239 10.1046/j.1524-4733.2003.00204.x

[47] Ramacciotti E, Gomes M, de Aguiar ET, Caiafa JS, de Moura LK, Araujo GR, et al (2006) A cost analysis of the treatment of patients with post-thrombotic syndrome in Brazil. Thromb Res 118: 699–704. 16417913 10.1016/j.thromres.2005.12.005

[48] Ten Cate-Hoek AJ, Toll DB, Buller HR, Hoes AW, Moons KG, Oudega R, et al (2009) Cost-effectiveness of ruling out deep venous thrombosis in primary care versus care as usual. J Thromb Haemost 7: 2042–2049. 10.1111/j.1538-7836.2009.03627.x 19793189

[49] Cv Zorgverzekeringen (2004) Handleiding voor kostenonderzoek. Methoden en standaard kostprijzen voor economische evaluaties in de gezondheidszorg: College voor zorgverzekeringen.

[50] Kind P, Hardman G, Macran S (1999) UK Population Norms for EQ-5D Discussion paper 172. Centre for Health Economics The University of York.

[51] Sullivan PW, Slejko JF, Sculpher MJ, Ghushchyan V (2011) Catalogue of EQ-5D scores for the United Kingdom. Med Decis Making 31: 800–804. 10.1177/0272989X11401031 21422468

[52] Roberts M, Russell LB, Paltiel AD, Chambers M, McEwan P, Krahn M (2012) Conceptualizing a model: a report of the ISPOR-SMDM Modeling Good Research Practices Task Force—2. Value Health 15: 804–811. 10.1016/j.jval.2012.06.016 22999129 PMC4207095

