# Supplementary material for: Sample Size Estimation for Non-Inferiority Trials: Frequentist Approach versus Decision Theory Approach
Source: PLoS One. 2015 Jun 15;10(6):e0130531. doi: 10.1371/journal.pone.0130531 (PMC4468148; doi:10.1371/journal.pone.0130531)
Supplement: S1 Appendix — (DOCX) [file pone.0130531.s001.docx]

**Supporting Information - Appendix**

***Model description***

A probabilistic state transition health economic decision model was developed to assess the lifetime consequences on costs and quality adjusted life years (QALYs) of the two therapies: individually tailored ECS therapy; and two years ECS therapy, after DVT. Patients after a first proximal DVT of the leg are included in the decision model.

The decision model consists of four mutually exclusive health states. Cohort simulation was used to evaluate the decision model: each cycle a hypothetical cohort of patients moves between the health states according to a set of transition probabilities. Cycle length is 6 months for the first two cycles, and 1 year for the cycles thereafter. The time horizon of the decision model is lifelong, because PTS is a chronic condition.

The decision model was built and analyzed in Microsoft Excel 2010.

***Model construction***

*Health states*

The decision model consists of 4 mutually exclusive health states: No PTS, Mild to moderate PTS, Severe PTS, and Death. States 1-3 are based on the diagnosis of PTS using the Villalta score, a scoring scale used to diagnose and rate post thrombotic complaints and signs, developed by Prandoni and colleagues.[[1](#_ENREF_1)] PTS is diagnosed if the Villalta score is ≥5 on two consecutive visits that are at least 3 months apart or a venous ulcer is present. PTS is classified as mild to moderate if the Villalta score is 5-14, and severe if the Villalta score is ≥15 or a venous ulcer is present.[[2](#_ENREF_2)] The final ‘absorbing’ state is ‘death’ or all-cause mortality.

*Strategies*

In the decision model two strategies are compared: (1) individually tailored ECS therapy, after the first 6 months duration of ECS therapy is based on the signs and symptoms of the individual patient and (2) two years ECS therapy, all patients are advised to wear the elastic compression stocking for a standard duration of two years after DVT. The difference between the two strategies was modeled by taking into account the differential impact on the probability of developing PTS, the costs of ECS (for stocking and home care for stocking application), and the disutility of ECS therapy.

*Model assumptions*

The following assumptions were adopted in this health economic decision model:

- After two years no additional patients develop PTS, since only very few patients develop PTS beyond two years after DVT.[[1](#_ENREF_1)]
- PTS incidence is equal in both strategies. This assumption is based on a management study in which individually tailored ECS therapy was applied in 125 patients after DVT, and appeared to generate a comparable PTS incidence as the active arm (two years ECS therapy) of previous RCTs.[[3](#_ENREF_3)]
- All patients without PTS (both strategies) stop ECS therapy at two years after DVT, if they have not stopped ECS therapy yet.
- All patients with PTS (mild to moderate or severe) continue ECS therapy for the rest of their lives.

*Transition probabilities*

The transition probabilities for the development of PTS for each cycle were derived from an RCT by Prandoni et al., in which two years ECS therapy was compared to no ECS therapy. The 6-month, 12-month, and 24-month probability to develop PTS was extracted from this study.[[1](#_ENREF_1)] PTS incidence was assumed to be equal for individually tailored ECS therapy and two years ECS therapy, based on a management study on individually tailored ECS therapy.[[3](#_ENREF_3)]. A relative risk (RR) parameter was incorporated in the decision model to represent the uncertainty of the development of PTS with individually tailored ECS therapy. A log normal distribution, with a mean of 1.000 and 95% confidence intervals (CI) ranging between 1.000 and 3.316 (the standard error of the LN(RR) is 0.612) is used. The RR parameter is multiplied with the probabilities of developing PTS after six months with individually tailored ECS therapy. The upper limit of the 95% CI corresponds with a 7.5% increase in the two year PTS incidence, representing the expected maximal increase in PTS incidence with individually tailored ECS therapy. Values of the parameter below 1 are rounded up to 1, because we assume that individually tailored ECS therapy does not lead to a decrease in PTS incidence. For individually tailored ECS therapy, the transition probabilities for taking off the stocking and compliance of ECS therapy after six and after twelve months were derived from the results of the management study.[[3](#_ENREF_3)] For two years ECS therapy, the transition probabilities concerning compliance of ECS therapy in the two years after DVT were derived from the RCT by Prandoni et al.[[1](#_ENREF_1)]

*Health effects*

Health-related quality of life in the form of utility scores were used as an outcome of the decision model. Utility scores provide a single index value for health status, ranging from 0 (death) to 1 (perfect health). Utility scores for the different health states were derived from literature. Patients who do not develop PTS were assumed to have an equal utility as the general population. Therefore, for the health state No PTS the utility was derived from age-specific EuroQol 5D (EQ5D) utilities from the general population.[[4](#_ENREF_4)] Since no data were available on utility scores for mild to moderate and severe PTS specifically, we used utilities of conditions which are very similar to PTS. Patients with mild to moderate PTS were assumed to have a utility comparable to patients with varicose veins of the lower extremity. Patients with severe PTS were assumed to have a utility comparable to patients with a chronic ulcer of the skin. The utilities of varicose veins of the lower extremity and chronic ulcer of the skin were derived from a catalogue of EQ5D utilities from the United Kingdom.[[5](#_ENREF_5)]

Since most patients experience ECS therapy as unpleasant, because stockings are itchy; warm; pinching; and difficult to apply, a disutility was assigned to ECS therapy in case PTS was not present. In literature no data were present to substantiate this disutility of ECS therapy, and therefore it was based on expert opinion.

*Costs*

A healthcare perspective was used. All costs were reported in Euros and price indices were used to convert the costs to the 2013 price level. As far as possible, costs were based on the Dutch manual for cost research.[[6](#_ENREF_6)] Costs of PTS were derived from literature, expert opinion, and the previously published AMUSE model.[[7-10](#_ENREF_7)]

***Model analysis***

Distributions were assigned to the input parameters and probabilistic sensitivity analysis with 5,000 iterations was used to generate expected outcomes. Future costs and effects were discounted using a discount rate of 4% for costs and 1.5% for (quality adjusted) life years, according to Dutch guidelines.[[11](#_ENREF_11)] For both therapies (individually tailored ECS therapy and two years ECS therapy) a model was built. Total costs and QALYs were calculated for each model. Cost-effectiveness acceptability curves were calculated, for different thresholds.

Value of information analyses were performed: expected value of perfect information (EVPI) and expected value of perfect parameter information (EVPPI), for an effective population of 25,000 patients, the total yearly incidence of DVT in the Netherlands [[12](#_ENREF_12)], over a lifetime of individually tailored ECS therapy of 10 years. A threshold of € 20,000 per QALY was used.

Furthermore, the optimal sample size of a future trial was evaluated, using the expected value of sampling information (EVSI) analysis and expected net benefit of sampling (ENBS). For different sample sizes (n=25, n=100, n=400, n=500, n=700, n=1000, n=1500, n=5000), two hundred possible trial results were simulated, using Monte Carlo simulation with 1000 iterations. Model parameters concerning the development of PTS and the relative risk parameter were updated with the simulated trial results. Subsequently the resulting EVPI per patient based on the simulated trial results was calculated. To calculate the updated population EVPI, the sample size of the trial was subtracted from the effective population, because the patients participating in the trial do not benefit from the additional information obtained in the trial. The EVSI is the prior EVPI (before update with simulated trial results) minus the new EVPI. Per sample size the two hundred EVSI results are averaged, to obtain the mean EVSI of that sample size. For the trial we assumed a fixed cost of € 10,000,- and a variable cost of € 5000,- per included patient. The ENBS was calculated by subtracting the trial costs from the EVSI. Sensitivity analyses were performed to explore the effect of changes in the size of the effective population on the EVSI and ENBS results.

**Table Appendix 1. Input parameters model**

| **Parameter** | **Mean** | **SE** | **Distribution** | **Ref** |
| --- | --- | --- | --- | --- |
| **Transition probabilities** |  |  |  |  |
| Probability Mild to moderate PTS after 6 months | 0.176 | 0.046 | Beta | [[1](#_ENREF_1)] |
| Probability Mild to moderate PTS after 12 months | 0.036 | 0.047 | Beta | [[1](#_ENREF_1)] |
| Probability Mild to moderate PTS after 24 months | 0.033 | 0.045 | Beta | [[1](#_ENREF_1)] |
| Probability Severe PTS after 6 months | 0.035 | 0.018 | Beta | [[1](#_ENREF_1)] |
| Probability Severe PTS after 12 months | 0.000 |  |  | [[1](#_ENREF_1)] |
| Probability Severe PTS after 24 months | 0.000 |  |  | [[1](#_ENREF_1)] |
| Uncertainty of probability PTS | 1.000 | 0.612* | Log normal | [[1](#_ENREF_1),[3](#_ENREF_3)] e.o. |
| Probability stocking off 0-6 months *- individually tailored ECS* | 0.197 | 0.035 | Beta | [[3](#_ENREF_3)] |
| Probability stocking off 6-12 months *- individually tailored ECS* | 0.488 | 0.046 | Beta | [[3](#_ENREF_3)] |
| Probability stocking off 12-24 months *- individually tailored ECS* | 0.485 | 0.051 | Beta | [[3](#_ENREF_3)] |
| Compliance ECS therapy 0-6 months *- 2 years ECS* | 0.018 | 0.007 | Beta | [[1](#_ENREF_1)] |
| Compliance ECS therapy 6-12 months *- 2 years ECS* | 0.035 | 0.014 | Beta | [[1](#_ENREF_1)] |
| Compliance ECS therapy off 12-24 months *- 2 years ECS* | 0.070 | 0.027 | Beta | [[1](#_ENREF_1)] |
| **Quality of life consequences** |  |  |  |  |
| No PTS | Age-dependent norm utility post DVT | | | [[4](#_ENREF_4)] |
| Disutility – stocking | 0.052 |  | Uniform | e.o. |
| Mild to moderate PTS – disutility | 0.117 | 0.050 | Beta | [[5](#_ENREF_5)] |
| Severe PTS – disutility | 0.218 | 0.040 | Beta | [[5](#_ENREF_5)] |
| **Costs of treatment and costs of consequences** |  |  |  |  |
| Costs stockings per year** | € 572 |  |  | [[7](#_ENREF_7),[9](#_ENREF_9)] |
| Costs Mild to moderate PTS 1st 6 months | € 273 |  |  | [[7](#_ENREF_7),[8](#_ENREF_8),[10](#_ENREF_10),[13](#_ENREF_13)] |
| Costs Mild to moderate PTS 2nd 6 months | € 183 |  |  | [[7](#_ENREF_7),[8](#_ENREF_8),[10](#_ENREF_10),[13](#_ENREF_13)] |
| Costs Mild to moderate PTS 2nd year on | € 183 |  |  | [[7](#_ENREF_7),[8](#_ENREF_8),[10](#_ENREF_10),[13](#_ENREF_13)] |
| Costs Severe PTS 1st 6 months | € 11,986 |  |  | [[7](#_ENREF_7),[8](#_ENREF_8),[10](#_ENREF_10),[13](#_ENREF_13)] |
| Costs Severe PTS 2nd 6 months | € 11,881 |  |  | [[7](#_ENREF_7),[8](#_ENREF_8),[10](#_ENREF_10),[13](#_ENREF_13)] |
| Costs Severe PTS 2nd year on | € 23,600 |  |  | [[7](#_ENREF_7),[8](#_ENREF_8),[10](#_ENREF_10),[13](#_ENREF_13)] |

*Standard error of LN(RR)
**Homecare for application of stockings included for those patients who need that.

DVT, deep vein thrombosis; ECS, elastic compression stocking; PTS, post thrombotic syndrome; QALY, quality adjusted life year

**References**

1. Prandoni P, Lensing AW, Prins MH, Frulla M, Marchiori A, et al. (2004) Below-knee elastic compression stockings to prevent the post-thrombotic syndrome: a randomized, controlled trial. Ann Intern Med 141: 249-256.

2. Kahn SR, Partsch H, Vedantham S, Prandoni P, Kearon C (2009) Definition of post-thrombotic syndrome of the leg for use in clinical investigations: a recommendation for standardization. J Thromb Haemost 7: 879-883.

3. Ten Cate-Hoek AJ, Ten Cate H, Tordoir J, Hamulyak K, Prins MH (2010) Individually tailored duration of elastic compression therapy in relation to incidence of the postthrombotic syndrome. J Vasc Surg 52: 132-138.

4. P. K, G. H, S. M (1999) UK Population Norms for EQ-5D Discussion paper 172. Centre for Health Economics The University of York.

5. Sullivan PW, Slejko JF, Sculpher MJ, Ghushchyan V (2011) Catalogue of EQ-5D scores for the United Kingdom. Med Decis Making 31: 800-804.

6. Oostenbrink JB, Bouwmans CAM, Koopmanschap MA, Rutten FF (2004) Handleiding voor kostenonderzoek

Methoden en standaard kostprijzen voor economische evaluaties

in de gezondheidszorg. Diemen: Instituut voor Medical Technology Assessment, Erasmus MC in opdracht van College voor zorgverzekeringen.

7. Ten Cate-Hoek AJ, Toll DB, Buller HR, Hoes AW, Moons KG, et al. (2009) Cost-effectiveness of ruling out deep venous thrombosis in primary care versus care as usual. J Thromb Haemost 7: 2042-2049.

8. Caprini JA, Botteman MF, Stephens JM, Nadipelli V, Ewing MM, et al. (2003) Economic burden of long-term complications of deep vein thrombosis after total hip replacement surgery in the United States. Value Health 6: 59-74.

9. Gelderblom GJ, Hagedoorn-Meuwissen EAV (2005) Kousen uittrekhulpmiddel Easy-Lever. Een onderzoek naar bruikbaarheid, effecten en belemmeringen, in opdracht van ZonMw.

10. Ramacciotti E, Gomes M, de Aguiar ET, Caiafa JS, de Moura LK, et al. (2006) A cost analysis of the treatment of patients with post-thrombotic syndrome in Brazil. Thromb Res 118: 699-704.

11. Oostenbrink JB, Koopmanschap MA, Rutten FF (2002) Standardisation of costs: the Dutch Manual for Costing in economic evaluations. Pharmacoeconomics 20: 443-454.

12. Naess IA, Christiansen SC, Romundstad P, Cannegieter SC, Rosendaal FR, et al. (2007) Incidence and mortality of venous thrombosis: a population-based study. J Thromb Haemost 5: 692-699.

13. zorgverzekeringen Cv (2004) Handleiding voor kostenonderzoek. Methoden en standaard kostprijzen voor economische evaluaties in de gezondheidszorg: College voor zorgverzekeringen.
